# Xeno-miRNA in Maternal-Infant Immune Crosstalk: An Aid to Disease Alleviation

**DOI:** 10.3389/fimmu.2020.00404

**Published:** 2020-03-24

**Authors:** Bjorn John Stephen, Nidhi Pareek, Mohd Saeed, Mohd Adnan Kausar, Safikur Rahman, Manali Datta

**Affiliations:** ^1^Department of Biosciences, Manipal University Jaipur, Jaipur, India; ^2^Department of Microbiology, School of Life Sciences, Central University of Rajasthan, Ajmer, India; ^3^Department of Biology, College of Sciences, University of Ha'il, Ha'il, Saudi Arabia; ^4^Department of Biochemistry, College of Medicines, University of Ha'il, Ha'il, Saudi Arabia; ^5^Department of Botany, Munshi Singh College, Babasaheb Bhimrao Ambedkar Bihar University, Muzaffarpur, India; ^6^Amity Institute of Biotechnology, Amity University Rajasthan, Jaipur, India

**Keywords:** breast milk, exosomes, xeno-miRNA, vertical transfer, micro RNA

## Abstract

Human milk is a complex liquid that contains multifaceted compounds which provide nutrition to infants and helps to develop their immune system. The presence of secretory immunoglobulins (IgA), leucocytes, lysozyme, lactoferrin, etc., in breast milk and their role in imparting passive immunity to infants as well as modulating development of an infant's immune system is well-established. Breast milk miRNAs (microRNAs) have been found to be differentially expressed in diverse tissues and biological processes during various molecular functions. Lactation is reported to assist mothers and their offspring to adapt to an ever-changing food supply. It has been observed that certain subtypes of miRNAs exist that are codified by non-human genomes but are still present in circulation. They have been termed as xeno-miRNA (XenomiRs). XenomiRs in humans have been found from various exogenous sources. Route of entry in human systems have been mainly dietary. The possibility of miRNAs taken up into mammalian circulation through diet, and thereby effecting gene expression, is a distinct possibility. This mechanism suggests an interesting possibility that dietary foods may modulate the immune strength of infants via highly specific post-transcriptional regulatory information present in mother's milk. This serves as a major breakthrough in understanding the fundamentals of nutrition and cross-organism communication. In this review, we elaborate and understand the complex crosstalk of XenomiRs present in mother's milk and their plausible role in modulating the infant immune system against infectious and inflammatory diseases.

## Introduction

Human milk is a complex liquid, containing multifaceted compounds that provide nutrition and help to develop infant's immune systems (1). The presence of secretory immunoglobulins (IgA), leucocytes, lysozyme, lactoferrin, etc., in breast milk and their role in imparting passive immunity to infants as well as modulating the of development of an infant's immune system is well-established. Furthermore, parental dietary signals can be passed to tens of succeeding generations. miRNA in milk appeared to be endogenous for mammary glands and employed as biomarkers to assess the performance and healthiness of glands during lactation (2). The abundance of variants in miRNAs in mammalian milk has led to curiosity over their influence on developing infant gastrointestinal and immune system, along with tissue-specific developmental functions. Over 600 miRNAs have been identified from human breast milk; a range of these possess immunological roles viz. regulation of T-cells and B-cells differentiation, while others have regulatory effects on various physiological and metabolic responses (3, 4).

Breast milk miRNAs exert differential expression in diverse tissues and modulate diverse molecular functions. Lactation has been considered as the first step in postnatal defense and immune-modulation, thus enabling adaptation in spite of an ever-changing food supply (5). The research illustrated that maternally-secreted miRNA plays regulatory roles, i.e., macromolecule biosynthesis and macromolecule metabolism in infants, and are also considered as contributors to the nutritional richness of breast milk. Human breast milk may be fractionated into cells, skim milk, and lipids. Each fraction contains varying concentrations of microRNA, with the highest in the cell and lipid fractions, and the lowest in skim milk. Transcriptome profiling revealed the inclusive presence of known and novel miRNA species in fat globules of human breast milk (6). An electropherogram profiling generated for miRNA using miRCURY-Biofluids kit indicate the following concentrations: 12.74 ± 24.44 ng/mL (lipid fraction), (2.17 ± 5.66 ng/mL) (skim milk), and 1,443 ± 3,448 ng/mL (cells) (3, 4).

Thus, we aim to confirm the significance of the correlative deviation of the relative composition of breast milk miRNA with maternal fat diet. Dietary comparisons viz. high fat-high carbohydrate and relative glucose-galactose resulted in notably increased detection of miR-67 and miR-27 in high fat intake as opposed to high carbohydrate intake. Deciphering human breast milk miRNomics may pave new avenues in infant disease alleviation and health augmentation.

### The Long and Short of miRNA

miRNAs are approximately 22-nucleotide-long non-coding small RNAs that mediate post-transcriptional gene silencing by binding to their mRNA targets. Discovered in 2004, miRNAs were involved in a wide range of biological processes including cell proliferation, cell differentiation, cell migration, disease initiation, and disease progression. The circulating miRNA are actively secreted by living cells or passively released during cell death. The miRNA profiles vary based on the type of cell/tissue or organ and are being suggested to serve as biomarkers for indication of different diseases.

The advent of next generation sequencing and genome-wide analysis has highlighted the existence of exogenous miRNAs. As a result of their stability and presence in bodily fluids, studies have hinted at an inter-individual epigenetic communication, whereby by having contact with their body fluids miRNA's released by one individual could have actions in another (7).

## XenomiRs and Their Horizontal Transfer in Humans

It has been observed that certain subtypes of miRNAs exist that are codified by non-human genomes but are present in the circulation. They have been termed as xeno-miRNA (XenomiRs). One of the earliest mentions of XenomiRs was reported by Zhang et al. (8), where they discovered exogenous plant miRNAs (miR168a in rice) existing in the sera and tissues of animals (8). XenomiRs in humans have been found from plant (9, 10), animal (11), and viral sources. Approximately 872 circulating miRNAs, belonging to 42 plant families, have been identified by genomic analysis screening and RNA sequencing, with 325 miRNAs fully annotated from the plant model *Arabidopsis thaliana* (12).

The route of entry of miRNAs in human systems has been mainly via the diet. Multiple studies based on cloning, RNA sequencing, dietary miRNA transfer experiments and analysis, as well as experimentation relating to the strong iodine-containing oxidizing agent, suggest that approximately 5% of the different types of miRNAs detected so far in the human serum are plant miRNAs containing a 2′ -*O*-methyl modified 3′ end making them resistant to oxidative degeneration (13). The prevalence of exogenous miRNAs in food has been evident irrespective of the intense amount of processing techniques. On comparison, food derived from animals demonstrate a higher incidence of the miRNA. The elusive question remains whether the XenomiRs are good or bad for humans? Evidence has cropped over the years that circulating XenomiRs may regulate the progression or alleviation of chronic maladies. A freely circulating candidate, miR2911 from honeysuckle, was found to have a singular effect of targeting Influenza A viruses (IAVs) and demonstrated anti-viral activity (14).

Extracellular miRNAs (EcmiRNA) when naked are rapidly degraded by extracellular RNases, and hence have been found to be ingested as either membrane vesicles or as stable macromolecular complexes. AGO-bound miRNAs (Argonaute-bound) have shown the capability of crossing gap junctions, thereby enabling modulation of gene expression in the host organism. Studies confirmed that dietary miRNA remains stable despite processing and a dedicated vegetarian-diet was found to be associated with an increase of plant miR-168 in GI (gastro-intestinal) mucosa, feces, and in fecal samples of colorectal cancer (15). Conversely, no changes in miR-21 were reported after 1-week meat-rich diet in feces. On the contrary, another study demonstrated red meat upregulates multiple tumor-associated miRNAs (miR-17-92 cluster and miR-21) (16).

**Figure 1:**
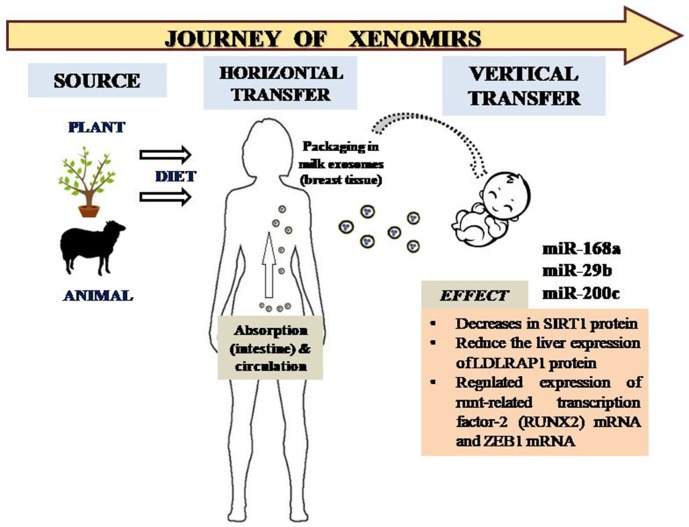
Transfer of XenomiRs from exogenous sources to humans and the subsequent journey into infants via packaged exosomes in milk. The XenomiRs affect specific targets in the human infant, thus effecting particular pathways corresponding to genome expression and regulation.

Validation of xenomiR in human milk generated an unexpected differential profile in human subjects with diminutive food habit fluctuations. A distinct demarcation could be observed in the miRNA profile of candidates with a vegetarian and non-vegetarian diet. Five plant food-derived miRNAs (miR166a, miR156a, miR157a, miR172a, and miR168a) were found to be significantly present in human breast milk. In contrast, quantification of the miRNA indicated a relatively higher concentration of miR166a and miR168a [whole milk] and miR156a and miR168a [exosomal fraction] in candidates with a non-vegetarian diet. miR168a was significantly lower in vegetarian candidates whereas miR172a was found to be prevalent in non-vegetarian ones (17). Milk-derived XenomiRs may have a different *modus operandi* in inter-species epigenetic communication. Surprisingly, homology has been found between animal and human miRNAs. Mir-155, present in the milk derived from bovine and human sources, was sequentially compared and observed to have a high percentage of sequence similarity (Figure 1). Hence, presence of such types of homologous miRNAs may generate evident physiological responses. Similar sequence similarities have also been cited in miR-21-5p and miR-30a-5p sequences in human and bovine samples (18). In addition to a varied diet, certain amounts of conservation of xenomiR present in the milk exosomes from different organisms has been observed. let-7 family members like let-7a-5p, let-7b-5p, and let-7f, as well as miR-148a, were found to be conserved in human, cow, pig, and panda species (19). Its uptake by human cell line [THP-1] was substantiated, thus suggesting that bovine milk exosomes containing miRNA can enter the circulatory system of humans (20). Time course analysis in a murine model indicated that there was distribution of exosomal miRNA in the liver, spleen, heart, and lungs even 3 h after intake (21). Galactose and sialo-galactose glycan modifications of the exosomal proteins have been implicated in the uptake of bovine miRNA in non-bovine species (22).

A study based on *C. elegans* indicated that uptake of miRNA from the human gut is mediated by two transport proteins, Sid-1 and Sid2. Sid-1, a multipass trans-membrane protein in stomach lumen, and Sid-2, an intestinal luminal transporter, channelizes dsRNA across the membrane. Sid-2 binds long dsRNA first for endocytosis, then the dsRNA is presented to Sid-1. An efficient uptake of dsRNA to the intestinal cell cytoplasm is facilitated by the activation of Sid-1 by Sid-2. Sid-1 is able to transfer small interfering RNA (siRNA) and miR-21 (23).

## Vertical Transfer of XenomiRs in Maternal Fetal Axis

Breast milk contains a high level of nutritional content and confers the first line of immunological security to the infant. miRNA have been detected in whey fractions of human breast milk, bovine milk, and rat milk. Profiling of 681 mature miRNAs present in human milk and fat and a comparative analysis revealed higher miRNA concentration in human milk and maternal peripheral blood mononuclear cells. Furthermore, the presence of epithelial cells in mature milk justified the predominant origin of human milk miRNAs from the mammary epithelium (4). Similarly, bovine milk miRNA was persistent in supernatant as well as in exosomes.

Exosomes, a subtype of extracellular vesicle of 30–120 nm diameter, constitute the highly bioactive fraction enabling cell-to-cell communication (24–27). Colostrum has a comparatively higher prevalence of range of miRNA as compared to mature milk (28). Tolerance of exosomes to acidic pH and RNases ensures their persistence in humans as well as in the infant gastro-intestinal (GI) tract, thus aiding functional effects.

Mother's milk is rich in miRNAs, whose expression is variable and dependent upon the developmental stage and nutritional requirements of the fetus. Although initial studies have indicated the miRNA transferred via milk was prone to digestion in the stomach (29), progressive studies have confirmed the uptake and their subsequent physiological post-transcriptional regulatory effects (30, 31). This serves as a major breakthrough in understanding the fundamentals of nutrition and cross-organism communication.

When monitored, the compositional variations of miRNA content indicated variation after 2 months of lactation, especially in breast milk for preterm babies (32). miR-16 and miR-21 have been observed to be stably expressed during a study period in raw pooled milk samples, whereas miR-146b showed a higher stability than miR-16 and miR-21 in lipids and skim milk fractions. Clock-time dependent variability in miR-16-5p emphasizes dynamic fluctuations variations in breast milk with the nourishment requirements of infants. miR-16 was found to be involved in the regulation of gastrointestinal rhythmicity of the lactating infant along with development of its coordination with maternal rhythms.

In a report by Zhao et al. (12), breast milk samples were analyzed and found to contain plant-derived miRNA with an 100% prevalence, whereas analysis of the sera indicated the presence of circulating exosomes with a 42.9% prevalence rate. Another group (33), has shown the consistent presence of plant XenomiRs in human plasma and exosomal fractions. Diet derived miRNA tend to have 2′-O-methylation and, hence, were easily distinguishable. Plant-derived miRNA are packed into microvescicles [MV] and released into the sera. These MVs with AGO2-associated exogenous miRNA are transported to other organs (8). Another prominent mechanism, especially in the case of miR2911, is association of the miRNA with proteinase K-resistant complex, enabling stable passage through the serum (34). Two plant-derived miRNAs, namely miR156a and miR168a, have been consistently found in human breast milk, thus confirming the uptake of plant XenomiRs by mammary glands. So as per the partial evidences generated, the passage of foreign miRNA is via the GI tract followed by encapsulation in exosomes, uptake of exosomes by the mammary gland, and subsequent transfer of the exosomes into the fetus via breast milk. The route of transfer of serum exosomes in breast milk, however, remains unclear. These human exosomes are resistant to proteinase K, pepsin, and pancreatin digestion but have the capability of being uptaken by macrophages (3, 34–36).

Studies have confirmed that transfer of human milk microRNA into infants enables alleviation of the immune system in infants, especially in the first 6 months of life. Milk miR-155 have been found to regulate T and B cells in the innate immune response of infants. As far as XenomiRs uptake, studies have been conducted on transgenic animals. However, there is definite gap in knowledge confirming the effect milk-derived XenomiRs have in infants. The presence of XenomiRs in soluble fractions of human breast milk ensures the uptake by the infant. Studies have already indicated that there is a vertical transfer of miRNAs across the fetal placental axis (37).

## Milk Nutrimiromics in Infant Immunity

Emerging research on mammary gland biology and bioactive molecules stated that miRNAs are one of the most important immunological agents. Studies have confirmed that transfer of human milk microRNA into infants enables alleviation of the immune system in infants, especially in the first 6 months of life. Milk miR-155 have been found to regulate T and B cells in the innate immune response of infants. As far as uptake of XenomiRs, validated studies have been conducted on transgenic animals. Analysis of lactation-related miRNA expression profiles in porcine milk exosomes during the lactation period (from newborn to 28 days after birth) revealed the presence of high numbers of immune-related miRNAs in colostrum (28). Thirteen unique immune-related miRNAs have observed to be present in breast milk exosomal vesicles (Table 1). miR-148a-3p was observed to be overexpressed during lactation period in porcine milk and observed to down-regulate the expression of DNA methyltransferase 3b (28, 55). However, the correlation between these and the subsequent impact on infant's growth and development is still unclear, and there is definite lacunae confirming milk-derived XenomiRs exercising their effect in infants. The presence of XenomiRs in soluble fractions of human breast milk ensures the uptake by the infant.

**Table 1:** Immune response of miRNAs expressed during lactation period.

**miRNA**	**Immune response**	**References**
miR-148a-3p	Potential biomarker for milk quality control Targets cancer-related TGIF-2^*^ and drug metabolism related PXR^*^ genes	(38–40)
miR-25-3p	Target potential inflammation mediator KLF4^*^, Vital for development of immune system	(41)
miR-30a-5p miR-30d-5p	Suppress the expression of p53 and its downstream target DRP1^*^ Promote cellular invasion and immunosuppression by direct targeting of GALNT7^*^, increased synthesis of immunosuppressive cytokine IL-10^*^	(42)
miR-182-5p	Promotes T-cell mediated immune responses Post-transcriptional inhibition of FOXO1^*^, a suppressor of resting T-lymphocyte proliferation	(43)
miR-200c-3p	Target ZEB1^*^, which regulates T-cell differentiation and CD4 expression, repress IL-2 production	(44, 45)
miR-574-3p	Probable biomarker for detecting and differentiating the major subtypes of diffuse large B-cell lymphomas	(46)
miR-30c-2-5p miR-30c-1-5p	Involved in oncogenesis and mmunosuppression	(42, 43)
miR-191-5p	Biomarker of colorectal cancer, primary effusion lymphoma, hepatocellular carcinoma	(47–49)
miR-375-3p	Regulation of epithelial properties for optimum epithelium-immune system cross-talk Modulation of TSLP^*^ expression	(50)
miR-21-5p	Negative regulation of TLR4^*^ by targeting tumor suppressor PDCD4^*^ Modulation of IL-12^*^	(51)
miR-27b-3p	Destabilization of mRNA abundance of lipopolysaccharide-mediated PPARγ^*^, associated with chronic inflammatory diseases	(52)
let-7a-1-5p	Regulation of IL-6 induced STAT3^*^ signaling	(53)
miR-168a	Inhibiting LDL receptor expression in mouse liver	(8)
mi-29b	Increase in expression of runt-related transcription factor 2 (*RUNX2*), of miR-29b, increased in blood mononuclear cells	(54)
miR166a, iR156a, miR157a, miR172a miR168a	Possible impact on several critical biological pathways in infant organism	(17)
miRNA148a	targets DNA methyltransferase 1 (DNMT1) suppressing transcription	(7)

* *TGIF-2, TGFB induced factor homeobox 2; PXR, pregnane x receptor; KLF4, Kruppel like factor 4; DRP1, Dynamin related protein 1; GALNT7, GalNActransferase 7; IL-10, Interleukin-10; FOXO1, forkhead box protein O1; ZEB1, zinc finger E-box-binding homeobox 1; TSLP, thymic stromal lymphopoietin; TLR4, Toll like receptor 4; PDCD4, programmed cell death protein 4; IL-12, interleukin 12; PPARγ, peroxisome proliferation activated receptor γ; STAT3, signal transducers and activators of transcription 3*.

Studies have already indicated the presence of XenomiRs in the umbilical cord and cord blood, and the possibility of vertical transfer of miRNAs across the fetal placental axis (56). Transport of miRNA from mother to fetus in vesicles, mainly lipid encapsulated, provides them enhanced stability to cross through various barriers, particularly gastrointestinal ones (57). miR155 is the first miRNA associated with the immune system (58). It is transferred to the fetus via placental axis to neonates, as well as via milk to infants, and plays a key role in the maturation of macrophages and dendritic cells into active phenotype through toll-like receptors (59). miR223 was reported to act as a differentiation factor for monocytes and is responsible for proliferation and activation of granulocytes (60, 61). LPS (lipopolysaccharide) and pro-inflammatory cytokines (TNF-α, IL-1β) appeared as stimulants of miR146a and 146b, which in turn negatively regulate an acute innate immune response. miR146a is mainly responsible for NF-kB a mediated inflammatory response and type 1 interferon induction and signaling (62–64). However, miR125b is transcriptionally down-regulated via LPS stimulation and plays a key role in maintaining the levels of TNF-α (65).

Another set of miRNA involved in natal adipogenesis are miRNA-30b, miRNA-378, and let-7a. These have been found both in colostrum and mature milk. Higher incidence of let-7a and miRNA-378 has been quite evident in colostrum as compared to mature milk (66). Furthermore, let-7a/b/f-5p and miR-148-3p *in vivo* regulates transcription factor NF-κB, resulting in a suppression of the immune response (19). miR-148a-3p, along with miR-30b-5p, miR-182-5p, and miR-200a-3p, has also been designated as major immune-related pre-miRNAs (3), modulating the expression of TGIF2, PXR, and DNMT3B. Furthermore, miR-30b-5p stimulates cellular invasion and immunosuppression, miR-182-5p facilitates T-cell mediated immune responses, and miR-200a-3p has been linked with Hodgkin lymphoma and oral cancers. Other miRNAs associated with pathological and immune responses include miR-29a-3p (target interferon-γ, suppresses immune response to intracellular pathogens), miR-141-3p (biomarker for colon cancer), miR-378-3p (molecular switch involved in breast cancer cell metabolism), and miR-146b-5p (NF-kB signaling in innate immune responses). Colostrum contain miR-181a and hsa-miR-223, whose targets tend to be T and granulocytes cell populations, and hence affect the developmental stage of adaptive immune response in infants (67). Necrotizing enterocolitis afflicts 1–5% of premature neonates and there is a marked oxidative stress associated in the intestinal epithelial compartment. miRNA-125b present in breast milk-derived exosomes exhibits a prospective role in decreasing cell toxicity by targeting TLR4-dependent regulation of p53 (68). A distinct correlation has been found between Hsa-miR-195-5p and HsamiR-191-5p and CD^4+^ T-cell counts.

## XenomiRs of Breast Milk and Their Effect

To date, few XenomiRs have been functionally annotated. Plant-specific miR-168a, obtained from *Magnifera indica*, is acquired by humans via ingestion, and propelled into circulation via the GI tract. *Magnifera Oleifera* miR-168a is a functional homolog of human miR-579. Transfection of miR-168a in hepatic cancer cell lines effects the expression of NAD-dependent deacetylase sirtuin-1(SIRT1) protein, a valid target of miR-579, further supporting this cross-kingdom regulation (69, 70). Sirt1 catalyzes the formation of unique metabolite O-acetyl-ADP ribose, which regulates the functioning of several transcription factors. Another instance of the regulatory action of miR-168a was found in the case of low-density lipoprotein receptor adapter protein 1 (LDLRAP1), involved in vesicular endocytosis, whereby expression of hepatic LDLRAP1 was down-regulated upon exposure to miR-168a. LDLRAP1 is one of the major transport proteins involved in cholesterol uptake by the cells (8, 54).

miR-29b and miR-200c found in bovine milk tends to target the runt-related transcription factor-2 (RUNX2) mRNA and zinc finger E-box-binding homeobox 1 (ZEB1) mRNA and, furthermore, no homeostatic maintenance of the depleted levels of these miRNAs were compensated by intracellular synthesis. RUNX2 is involved in osteoblast differentiation and bone mineralization, whereas ZEB1 modulates the expression of IL2 *in vivo* (Figure 1).

## Conclusions

The potential implication of XenomiRs affecting and inducing epigenetic modification in humans is vast. There is a marked absence of validated data regarding the vertical transfer of xenomiR to infants. With the advent of state of the art technology, procedures have emerged which can distinguish the dietary miRNAs and endogenous miRNAs even at minimal concentrations and their progressive packaging in the milk exosomes. Enrichment of the functionally annotated XenomiRs has taken a major leap via *in silico* method. Techniques like RNase H-dependent PCR (rhPCR), combined with exhaustive high throughput sequencing, may further add to the plethora of knowledge pertaining to XenomiRs (71).

## Author Contributions

MD and SR conceived the idea. BS, MD, and NP contributed to writing of the manuscript. MD and BS prepared figures and/or tables. SR, MS, and MK reviewed drafts of the paper and approved the final draft.

### Conflict of Interest

The authors declare that the research was conducted in the absence of any commercial or financial relationships that could be construed as a potential conflict of interest.
